# Supplementary material for: Identification of circulating miRNAs as fracture-related biomarkers
Source: PLoS One. 2024 May 31;19(5):e0303035. doi: 10.1371/journal.pone.0303035 (PMC11142570; doi:10.1371/journal.pone.0303035)
Supplement: S1 Table — (DOCX) [file pone.0303035.s001.docx]

**Supplementary information** to "Identification of circulating miRNAs as fracture-related biomarkers"

**Table S1. Summary of subjects enrolled in the study.**

| **Donor info** | | | | | **Fracture patients' serum collection info** | | |
| --- | --- | --- | --- | --- | --- | --- | --- |
| **Donor #** | **Age**  **(yrs)** | **Sex**  **(M/F)** | **Healthy control / Fracture (location, type)** | **Fracture treatment** | **Early sample y/n** | **Late sample y/n** | **Very late sample y/n** |
| 1 | 28 | M | Fracture  (Clavicula) | Conservative | y | y | y |
| 2 | 59 | M | Fracture  (Olecranon, Schatzker A) | Surgical | y | n | y |
| 5 | 39 | F | Fracture  (Fibula, Weber B) | Surgical | n | y | n |
| 6 | 22 | M | Fracture  (Olecranon, Schatzker B) | Surgical | y | n | n |
| 7 | 22 | M | Fracture  (Tibia, C2.2) | Surgical | n | y | n |
| 8 | 43 | F | Fracture  (Tibial plateau dislocation fracture) | Surgical | y | y | y |
| 9 | 19 | F | Fracture  (Ankle, Weber C) | Surgical | y | n | y |
| 10 | 36 | F | Fracture  (Ankle, Weber C) | Surgical | y | n | n |
| 11 | 27 | M | Fracture  (Distal radius, Smith) | Surgical | y | n | y |
| 12 | 18 | F | Fracture  (Clavicula) | Surgical | y | n | n |
| 14 | 27 | M | Fracture  (Distal radius, B1.2) | Surgical | y | n | y |
| 17 | 43 | F | Fracture  (Ankle, Weber B) | Surgical | y | n | n |
| 19 | 23 | M | Healthy control | - | - | - | - |
| 20 | 27 | M | Healthy control | - | - | - | - |
| 21 | 25 | M | Healthy control | - | - | - | - |
| 22 | 28 | M | Healthy control | - | - | - | - |
| 23 | 24 | M | Healthy control | - | - | - | - |
| 24 | 21 | M | Healthy control | - | - | - | - |
| 25 | 29 | M | Healthy control | - | - | - | - |
| 26 | 29 | M | Healthy control | - | - | - | - |
| 27 | 25 | M | Healthy control | - | - | - | - |
| 28 | 27 | M | Healthy control | - | - | - | - |
