# Supplementary material for: Identification of circulating miRNAs as fracture-related biomarkers
Source: PLoS One. 2024 May 31;19(5):e0303035. doi: 10.1371/journal.pone.0303035 (PMC11142570; doi:10.1371/journal.pone.0303035)
Supplement: S2 Table — (DOCX) [file pone.0303035.s002.docx]

**Table S2. qPCR assay details**

| **Gene/miRNA ID** | **Forward sequence (or assay ID)** | **Reverse sequence** | **Probe sequence^‡^** |
| --- | --- | --- | --- |
| *BCL2* | Hs00608023_m1* |  |  |
| *COL1A1* | 5'-CCC TGG AAA GAA TGG AGA TGA T-3' | 5'-ACT GAA ACC TCT GTG TCC CTT CA-3' | 5'-CGG GCA ATC CTC GAG CAC CCT -3' |
| *IBSP* | Hs00173720_m1* |  |  |
| *ICAM1* | Hs00164932_m1* |  |  |
| *PDE1C* | Hs01095682_m1* |  |  |
| *PPARG* | Hs00234592_m1* |  |  |
| *RPLP0^#^* | 5'-TGG GCA AGA ACA CCA TGA TG-3' | 5'-CGG ATA TGA GGC AGC AGT TTC-3' | 5'-AGG GCA CCT GGA AAA CAA CCC AGC-3' |
| RUNX2 | 5'-AGC AAG GTT CAA CGA TCT GAG AT-3' | 5'-TTT GTG AAG ACG GTT ATG GTC AA-3' | 5'-TGA AAC TCT TGC CTC GTC CAC TCC G-3' |
| *SOX9* | Hs00165814_m1* |  |  |
| *SPARC* | Hs00234160_m1* |  |  |
| *SPP1* | 5'-CTC AGG CCA GTT GCA GCC-3' | 5'-CAA AAG CAA ATC ACT GCA ATT CTC-3' | 5'-AAA CGC CCA AGG AAA ACT CAC TAC C-3' |
| hsa-miR-3195 | YP02100869** |  |  |
| hsa-miR-7704 | YP02110126** |  |  |
| hsa-miR-31-5p | YP00204236** |  |  |
| hsa-miR-125a-5p | YP00204339** |  |  |
| hsa-miR-335-5p | YP02119293** or 478324_mir* |  |  |
| hsa-miR-23b-3p | YP00204790** |  |  |
| hsa-miR-411-5p | YP00204531** |  |  |
| hsa-miR-127-3p | YP00204048** |  |  |
| hsa-miR-26b-5p | YP00204172** |  |  |
| hsa-miR-103a-3p | YP00204063** |  |  |
| hsa-miR-222-3p | YP00204551** |  |  |
| hsa-miR-16-5p | YP00205702** |  |  |
| hsa-miR-193a-5p | YP00204665** or 477954_mir* |  |  |
| hsa-miR-423-3p | YP00204488** |  |  |
| hsa-miR-6821-5p | YP02108669** |  |  |
| hsa-miR-1246 | 483023_mir* |  |  |

* TaqMan assay (Thermo Fisher). 5' modification: FAM. 3' modification: NFQ-MGB.

** miRCURY Assay cat. no. Detection by SYBR green chemistry.

**^‡^** 5' modification: FAM. 3' modification: TAMRA.

*^#^* Reference gene.
