# Supplementary material for: Identification of circulating miRNAs as fracture-related biomarkers
Source: PLoS One. 2024 May 31;19(5):e0303035. doi: 10.1371/journal.pone.0303035 (PMC11142570; doi:10.1371/journal.pone.0303035)
Supplement: S3 Table — (DOCX) [file pone.0303035.s003.docx]

**Table S3. Full list of differentially regulated miRNA at day 7 in conditioned medium (osteo vs ctrl), sorted by Log2FC.**

| **miRNA** | **log2FC** | **PValue** | **FDR** |
| --- | --- | --- | --- |
| hsa-miR-6821-5p | 5.763107167 | 2.15E-05 | 0.000345688 |
| hsa-miR-4444 | 5.545712077 | 0.003589977 | 0.019551567 |
| hsa-miR-3195 | 4.880900438 | 3.68E-18 | 1.30E-15 |
| hsa-miR-1246 | 4.770853625 | 2.65E-14 | 4.69E-12 |
| hsa-miR-1273h-5p | 4.287772081 | 0.020481973 | 0.077134239 |
| hsa-miR-1273g-3p | 4.170283665 | 0.002106959 | 0.013318989 |
| hsa-miR-612 | 3.821952265 | 2.67E-07 | 7.26E-06 |
| hsa-miR-7704 | 3.693096455 | 2.19E-06 | 4.57E-05 |
| hsa-miR-6859-5p | 3.568034773 | 0.010466625 | 0.046314816 |
| hsa-miR-3178 | 3.540938362 | 0.01735591 | 0.068266579 |
| hsa-miR-3182 | 3.51528224 | 7.80E-05 | 0.000919982 |
| hsa-miR-3168 | 3.502892671 | 0.029376313 | 0.099992448 |
| hsa-miR-619-5p | 3.136599404 | 9.50E-05 | 0.000989627 |
| hsa-miR-4792 | 2.786874149 | 0.00032209 | 0.002780969 |
| hsa-miR-324-5p | 2.487032229 | 4.65E-05 | 0.000626603 |
| hsa-miR-3679-5p | 2.142086002 | 0.010797123 | 0.047187426 |
| hsa-miR-3187-3p | 2.007589222 | 0.02693074 | 0.095334819 |
| hsa-miR-423-3p | 1.946536223 | 1.43E-07 | 4.23E-06 |
| hsa-miR-365b-5p | 1.928913577 | 2.34E-05 | 0.000360363 |
| hsa-miR-193a-5p | 1.905514206 | 7.45E-08 | 2.40E-06 |
| hsa-miR-2110 | 1.67142737 | 0.047107318 | 0.13557716 |
| hsa-miR-34c-5p | 1.622048662 | 0.002448139 | 0.015204234 |
| hsa-miR-296-3p | 1.601069072 | 0.002962922 | 0.017194661 |
| hsa-miR-27a-5p | 1.543402032 | 0.002640352 | 0.015578074 |
| hsa-miR-484 | 1.484567568 | 0.000126927 | 0.001283775 |
| hsa-miR-193b-5p | 1.417169074 | 0.009935602 | 0.044521558 |
| hsa-miR-423-5p | 1.387776934 | 6.50E-05 | 0.000803276 |
| hsa-miR-92a-1-5p | 1.379981131 | 0.028105115 | 0.097541281 |
| hsa-miR-671-5p | 1.348213191 | 0.001968358 | 0.012903682 |
| hsa-miR-1270 | 1.269697616 | 0.036594516 | 0.117767806 |
| hsa-miR-138-5p | 1.032281694 | 0.002049705 | 0.013192644 |
| hsa-miR-370-3p | 1.01795903 | 0.00043128 | 0.003635074 |
| hsa-miR-485-5p | 1.010733736 | 0.01635511 | 0.067322198 |
| hsa-miR-19b-3p | 0.986955206 | 0.003577932 | 0.019551567 |
| hsa-miR-339-3p | 0.984968335 | 0.039390834 | 0.125624823 |
| hsa-miR-625-3p | 0.98283359 | 0.018528562 | 0.071984291 |
| hsa-miR-483-5p | 0.896138184 | 0.045320044 | 0.13557716 |
| hsa-miR-92b-5p | 0.891511909 | 0.045473945 | 0.13557716 |
| hsa-miR-493-3p | 0.877817409 | 0.003734422 | 0.020030083 |
| hsa-miR-378a-3p | 0.821203899 | 0.017315106 | 0.068266579 |
| hsa-miR-654-3p | 0.798064576 | 0.001779326 | 0.012350614 |
| hsa-miR-7-5p | 0.770633415 | 0.021608412 | 0.079681019 |
| hsa-miR-660-5p | 0.715969624 | 0.017279791 | 0.068266579 |
| hsa-miR-151a-3p | 0.695375584 | 0.00774282 | 0.037547373 |
| hsa-miR-199b-5p | 0.679072921 | 0.044671304 | 0.13557716 |
| hsa-miR-574-3p | 0.607335081 | 0.036452216 | 0.117767806 |
| hsa-let-7g-5p | -0.624951258 | 0.019249935 | 0.073273945 |
| hsa-miR-100-5p | -0.661883429 | 0.046349454 | 0.13557716 |
| hsa-miR-382-5p | -0.684005683 | 0.014841526 | 0.061810592 |
| hsa-miR-21-5p | -0.691231143 | 0.007081879 | 0.03530965 |
| hsa-miR-199a-5p | -0.711709285 | 0.009002548 | 0.042492027 |
| hsa-miR-320a | -0.742943527 | 0.009581236 | 0.044521558 |
| hsa-miR-365a-3p | -0.744870303 | 0.044159365 | 0.13557716 |
| hsa-miR-361-5p | -0.770625529 | 0.014132731 | 0.060276951 |
| hsa-miR-500a-3p | -0.800533407 | 0.040034199 | 0.126536665 |
| hsa-miR-143-3p | -0.807872123 | 0.00340715 | 0.019144941 |
| hsa-miR-10a-5p | -0.809714101 | 0.007515374 | 0.036950589 |
| hsa-miR-361-3p | -0.81099271 | 0.045894782 | 0.13557716 |
| hsa-miR-379-5p | -0.836495467 | 0.014698393 | 0.061810592 |
| hsa-miR-151a-5p | -0.867203329 | 0.030518851 | 0.102272822 |
| hsa-miR-27b-3p | -0.912303743 | 0.000310158 | 0.002744896 |
| hsa-let-7d-5p | -0.919229853 | 0.004395828 | 0.022884164 |
| hsa-miR-140-3p | -0.953974833 | 0.002575812 | 0.015454871 |
| hsa-miR-425-5p | -0.974626555 | 0.001936374 | 0.012903682 |
| hsa-miR-374a-5p | -1.082024394 | 0.026758564 | 0.095334819 |
| hsa-miR-99a-5p | -1.121214193 | 0.018707782 | 0.071984291 |
| hsa-let-7b-3p | -1.124700463 | 0.047067566 | 0.13557716 |
| hsa-miR-21-3p | -1.151617752 | 0.001166221 | 0.009174268 |
| hsa-miR-30e-3p | -1.196284239 | 8.47E-05 | 0.000937335 |
| hsa-miR-136-3p | -1.329538382 | 0.006250646 | 0.03161041 |
| hsa-miR-34c-3p | -1.330543713 | 0.042901851 | 0.133221538 |
| hsa-miR-222-3p | -1.333759675 | 2.81E-05 | 0.000414475 |
| hsa-miR-132-5p | -1.398745466 | 0.026334273 | 0.095125843 |
| hsa-miR-26b-5p | -1.412174109 | 4.42E-06 | 8.23E-05 |
| hsa-miR-26a-5p | -1.415866231 | 8.14E-05 | 0.000929277 |
| hsa-miR-664a-3p | -1.425257089 | 0.020805798 | 0.077528974 |
| hsa-miR-485-3p | -1.428255869 | 0.002557189 | 0.015454871 |
| hsa-miR-221-3p | -1.442721995 | 0.001662159 | 0.011768086 |
| hsa-miR-502-3p | -1.472513863 | 0.00186388 | 0.012688724 |
| hsa-miR-181a-5p | -1.499488385 | 0.001457459 | 0.010839717 |
| hsa-miR-210-3p | -1.521485256 | 0.00459277 | 0.023562909 |
| hsa-miR-181a-3p | -1.523011751 | 0.001318511 | 0.010146798 |
| hsa-miR-424-3p | -1.547795406 | 0.030624065 | 0.102272822 |
| hsa-miR-99b-5p | -1.552776481 | 0.000189767 | 0.001815612 |
| hsa-miR-127-3p | -1.598472931 | 2.57E-06 | 5.06E-05 |
| hsa-let-7i-5p | -1.604200193 | 8.93E-10 | 6.32E-08 |
| hsa-miR-335-5p | -1.606582844 | 2.55E-08 | 1.29E-06 |
| hsa-miR-27a-3p | -1.64064055 | 8.92E-05 | 0.000956539 |
| hsa-miR-142-3p | -1.722740961 | 0.046165546 | 0.13557716 |
| hsa-miR-628-5p | -1.742929293 | 0.000970277 | 0.007806317 |
| hsa-miR-214-5p | -1.765720844 | 0.008346551 | 0.039928093 |
| hsa-miR-125b-5p | -1.800838791 | 6.15E-08 | 2.18E-06 |
| hsa-miR-4454 | -1.820893261 | 0.000504081 | 0.004149878 |
| hsa-miR-455-3p | -1.829754656 | 0.016944021 | 0.068266579 |
| hsa-miR-331-3p | -1.881179313 | 0.00991735 | 0.044521558 |
| hsa-let-7f-5p | -1.912141043 | 9.65E-06 | 0.000162623 |
| hsa-miR-411-5p | -1.91218103 | 5.99E-08 | 2.18E-06 |
| hsa-miR-1180-3p | -1.92720331 | 0.001469792 | 0.010839717 |
| hsa-miR-103a-3p | -1.96506157 | 4.98E-06 | 8.82E-05 |
| hsa-miR-98-5p | -1.99682996 | 0.000259102 | 0.002383955 |
| hsa-miR-23b-3p | -2.031172699 | 4.78E-08 | 2.12E-06 |
| hsa-let-7a-5p | -2.039578811 | 7.51E-07 | 1.77E-05 |
| hsa-miR-7977 | -2.046430244 | 0.034675668 | 0.114721368 |
| hsa-miR-454-3p | -2.051198928 | 4.78E-05 | 0.000626603 |
| hsa-miR-181a-2-3p | -2.051990031 | 3.21E-05 | 0.000454378 |
| hsa-miR-125a-5p | -2.096759208 | 1.47E-08 | 8.65E-07 |
| hsa-let-7e-5p | -2.163766577 | 4.76E-07 | 1.20E-05 |
| hsa-miR-204-5p | -2.208000545 | 0.041309683 | 0.129412637 |
| hsa-miR-1179 | -2.378800501 | 0.023982727 | 0.087524591 |
| hsa-miR-126-3p | -2.419938895 | 0.004060291 | 0.021452878 |
| hsa-miR-335-3p | -2.787285921 | 1.24E-06 | 2.75E-05 |
| hsa-miR-548j-5p | -2.874233568 | 0.012275377 | 0.052993699 |
| hsa-miR-190a-5p | -3.312643599 | 1.24E-10 | 1.10E-08 |
| hsa-miR-181d-5p | -3.32890729 | 0.000262639 | 0.002383955 |
| hsa-miR-31-5p | -3.915725769 | 3.92E-11 | 4.63E-09 |
| hsa-miR-942-5p | -6.402371727 | 0.009906694 | 0.044521558 |
| hsa-miR-3065-5p | -6.703872261 | 0.003240764 | 0.018503716 |
| hsa-miR-374a-3p | -6.980537932 | 0.001573785 | 0.011369793 |
| hsa-miR-136-5p | -7.436706639 | 0.000133185 | 0.001309652 |
| hsa-miR-362-5p | -7.541905831 | 6.58E-05 | 0.000803276 |
