# Supplementary material for: Identification of circulating miRNAs as fracture-related biomarkers
Source: PLoS One. 2024 May 31;19(5):e0303035. doi: 10.1371/journal.pone.0303035 (PMC11142570; doi:10.1371/journal.pone.0303035)
Supplement: S4 Table — (DOCX) [file pone.0303035.s004.docx]

**Table S4. Full list of differentially regulated miRNA at day 15 in conditioned medium (osteo vs ctrl), sorted by Log2FC.**

| **miRNA** | **log2FC** | **PValue** | **FDR** |
| --- | --- | --- | --- |
| hsa-miR-2682-5p | 4.445690091 | 0.026239421 | 0.423954849 |
| hsa-miR-138-1-3p | 3.005870927 | 0.006302328 | 0.267555543 |
| hsa-miR-549a | 2.536832119 | 0.033417375 | 0.423954849 |
| hsa-miR-590-3p | 1.969163374 | 0.033571085 | 0.423954849 |
| hsa-miR-199b-5p | 1.820899724 | 0.000322987 | 0.036903638 |
| hsa-miR-619-5p | 1.577516643 | 0.023375252 | 0.423954849 |
| hsa-miR-4792 | 1.570147927 | 0.009528132 | 0.323956502 |
| hsa-miR-1262 | 1.52395625 | 0.025689009 | 0.423954849 |
| hsa-miR-378a-3p | 1.391275858 | 0.00033397 | 0.036903638 |
| hsa-miR-107 | 1.303804897 | 0.032128129 | 0.423954849 |
| hsa-miR-146a-5p | 1.244151429 | 0.003986018 | 0.238871008 |
| hsa-miR-1246 | 1.224240271 | 0.019248245 | 0.400425981 |
| hsa-miR-320d | 1.117006869 | 0.010268546 | 0.324192671 |
| hsa-miR-29b-3p | 1.052170547 | 0.018040233 | 0.39868915 |
| hsa-miR-99a-5p | 1.006424445 | 0.013645326 | 0.35511929 |
| hsa-miR-30a-5p | 1.005224042 | 0.003125719 | 0.230261276 |
| hsa-miR-10a-3p | 0.992573238 | 0.039432758 | 0.44690459 |
| hsa-miR-29a-3p | 0.955535678 | 0.004323457 | 0.238871008 |
| hsa-miR-125b-2-3p | 0.910938617 | 0.033416832 | 0.423954849 |
| hsa-miR-320c | 0.897897184 | 0.030819233 | 0.423954849 |
| hsa-miR-19b-3p | 0.872446603 | 0.026001793 | 0.423954849 |
| hsa-miR-320b | 0.868178215 | 0.028673335 | 0.423954849 |
| hsa-miR-30a-3p | 0.817146086 | 0.014036435 | 0.35511929 |
| hsa-miR-34a-5p | 0.797245365 | 0.019930705 | 0.400425981 |
| hsa-miR-532-5p | 0.728212564 | 0.030266262 | 0.423954849 |
| hsa-miR-155-5p | -0.675068938 | 0.044379513 | 0.45618011 |
| hsa-miR-125b-5p | -0.896534968 | 0.017517153 | 0.39868915 |
| hsa-miR-31-5p | -1.101585331 | 0.0476059 | 0.4782229 |
| hsa-miR-203a | -1.192816832 | 0.031098319 | 0.423954849 |
| hsa-miR-335-5p | -1.640067787 | 2.18927E-05 | 0.009676594 |
| hsa-miR-30e-5p | -2.056691643 | 0.040600302 | 0.448633337 |
| hsa-miR-542-3p | -2.308391387 | 0.044145171 | 0.45618011 |
| hsa-miR-3180-5p | -2.32980567 | 0.039015708 | 0.44690459 |
| hsa-miR-31-3p | -2.581571579 | 0.038733939 | 0.44690459 |
| hsa-miR-181c-3p | -2.651640581 | 0.036230249 | 0.444826941 |
| hsa-miR-184 | -2.687819831 | 0.043926674 | 0.45618011 |
| hsa-miR-23b-5p | -3.068114285 | 0.008901975 | 0.323956502 |
| hsa-miR-3180-3p | -3.422260708 | 0.00585791 | 0.267555543 |
| hsa-miR-142-3p | -3.907192106 | 0.022764366 | 0.423954849 |
| hsa-miR-205-5p | -3.91919681 | 0.006658622 | 0.267555543 |
| hsa-miR-26a-2-3p | -4.44949316 | 0.014461872 | 0.35511929 |
| hsa-miR-1228-3p | -4.488152369 | 0.013689219 | 0.35511929 |
| hsa-miR-142-5p | -7.400316309 | 0.001034472 | 0.091447368 |
| hsa-miR-223-3p | -8.726591274 | 6.55303E-05 | 0.014482204 |
