# Supplementary figures and images for: Identification of circulating miRNAs as fracture-related biomarkers
Source: PLoS One. 2024 May 31;19(5):e0303035. doi: 10.1371/journal.pone.0303035 (PMC11142570; doi:10.1371/journal.pone.0303035)

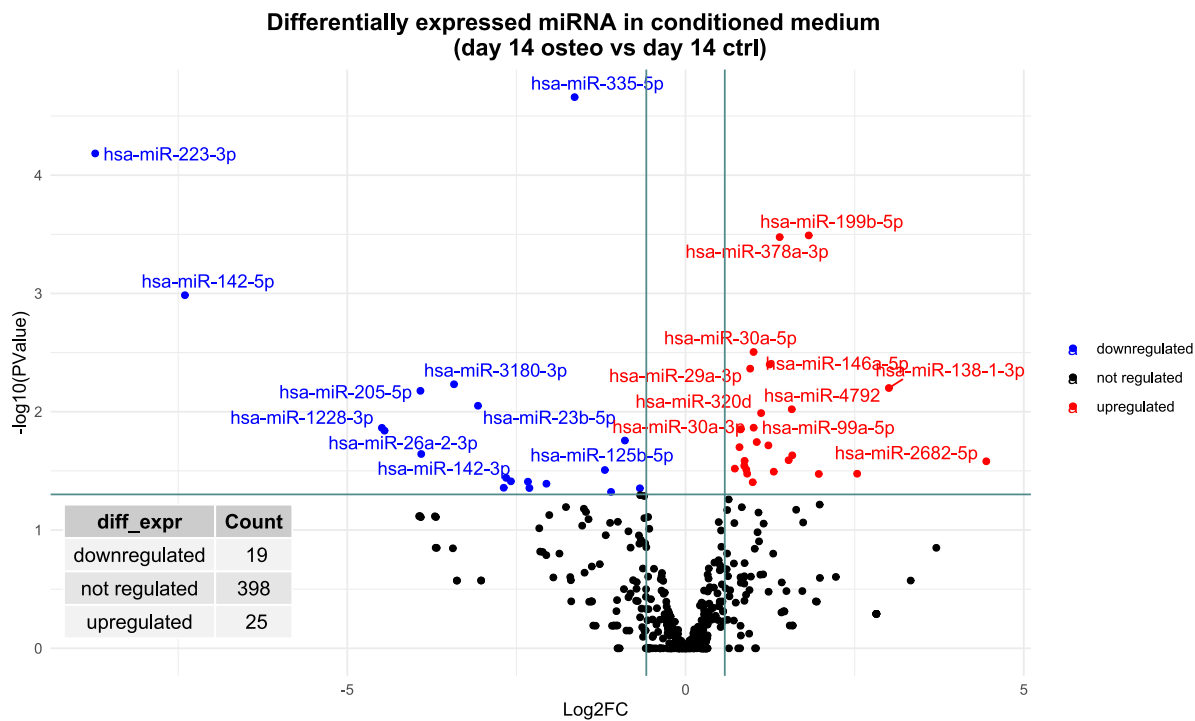

Supplement: S1 Fig — In volcano plots, the x-axis reports the log2 fold-change between osteo and ctrl (Log2FC), while the y-axis represents the -log10 of the p-value. Thresholds are set at 0.58 of log2FC (corresponding to an absolute 1.5-fold-change value) and to a -log10 p-value of 1.3 (corresponding to p-value of 0.05). (PDF) [file pone.0303035.s005.pdf]

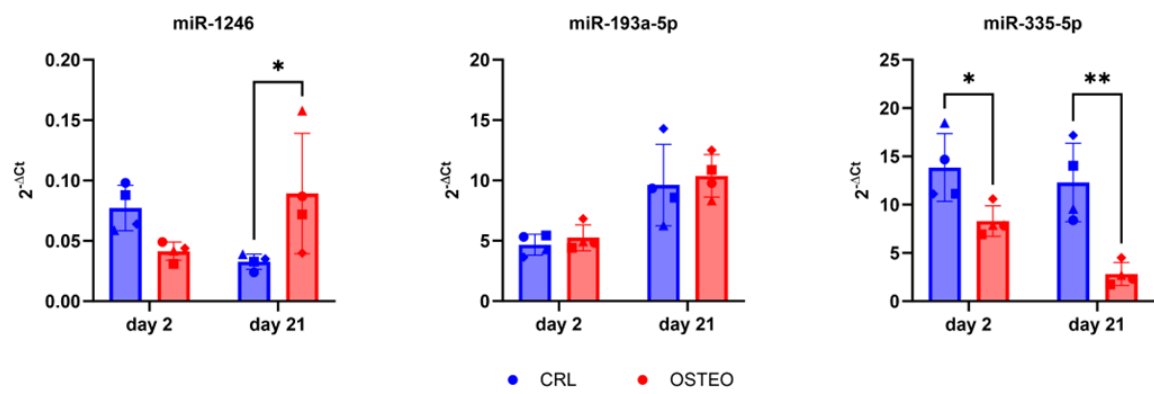

Supplement: S3 Fig — *p<0.05. **p<0.01. (PDF) [file pone.0303035.s007.pdf]
