# Supplementary material for: Identification of circulating miRNAs as fracture-related biomarkers
Source: PLoS One. 2024 May 31;19(5):e0303035. doi: 10.1371/journal.pone.0303035 (PMC11142570; doi:10.1371/journal.pone.0303035)
Supplement: S2 Fig — A) day 3 fracture serum samples versus controls. B) day 5–12 fracture serum samples versus controls. C) day 19–56 fracture serum samples versus controls. (PDF) [file pone.0303035.s006.pdf]

A)

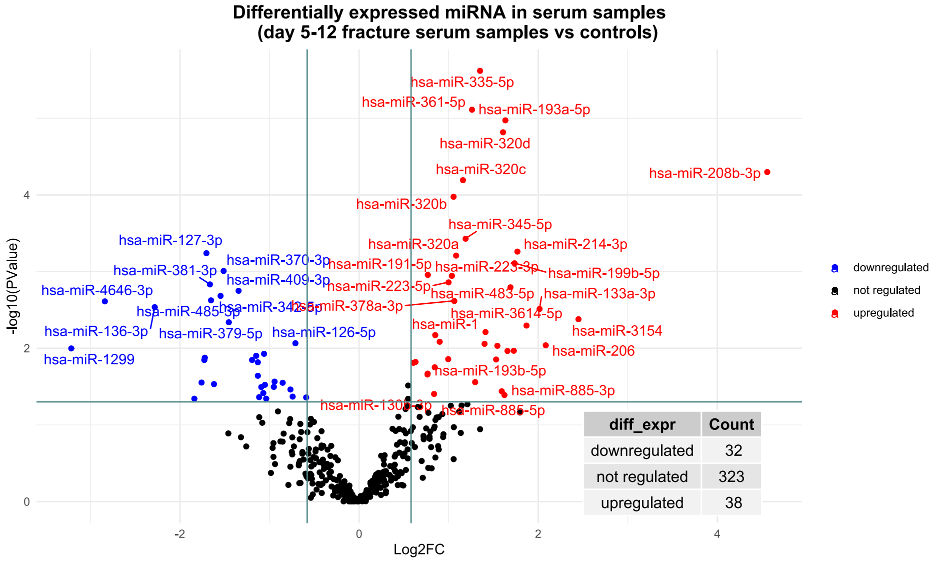

Differentially expressed miRNA in serum samples
(day 5-12 fracture serum samples vs controls)
hsa-miR-335-5p
hsa-miR-361-5p
hsa-miR-193a-5p
hsa-miR-320d
hsa-miR-320c
hsa-miR-208b-3p
hsa-miR-320b
hsa-miR-345-5p
hsa-miR-127-3p
hsa-miR-320a
hsa-miR-214-3p
hsa-miR-370-3p
hsa-miR-191-5p
hsa-miR-381-3p
hsa-miR-199b-5p
hsa-miR-409-3p
hsa-miR-4646-3p
hsa-miR-223-5p
hsa-miR-483-5p
hsa-miR-133a-3p
hsa-miR-378a-3p
hsa-miR-3614-5p
hsa-miR-1
hsa-miR-136-3p
hsa-miR-379-5p
hsa-miR-126-5p
hsa-miR-3154
hsa-miR-206
hsa-miR-1299
hsa-miR-193b-5p
hsa-miR-885-3p
hsa-miR-885-5p
diff_expr
Count
downregulated
32
not regulated
323
upregulated
38
downregulated
not regulated
upregulated
-log10(PValue)
Log2FC

B)

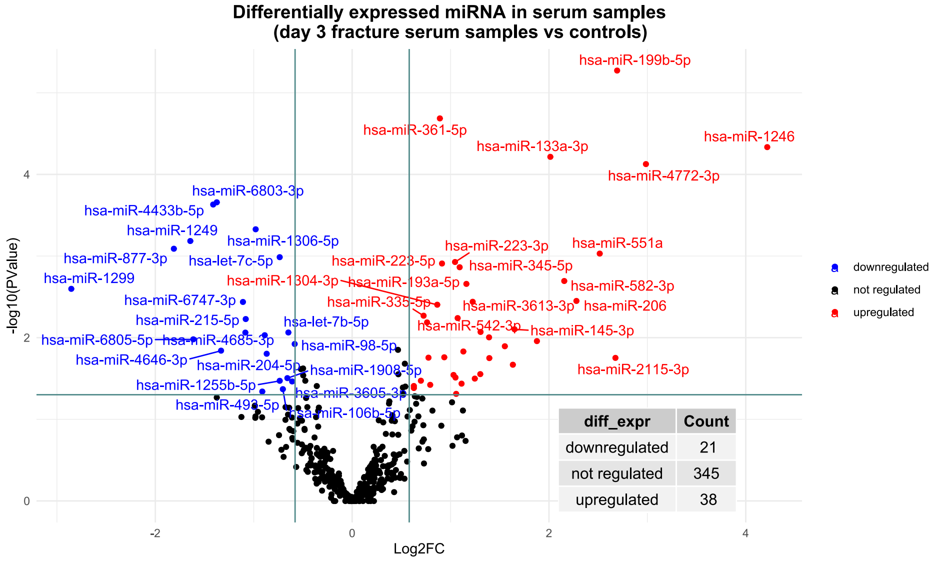

Differentially expressed miRNA in serum samples
(day 3 fracture serum samples vs controls)
hsa-miR-199b-5p
hsa-miR-361-5p
hsa-miR-1246
hsa-miR-133a-3p
hsa-miR-4772-3p
hsa-miR-6803-3p
hsa-miR-4433b-5p
hsa-miR-1249
hsa-miR-1306-5p
hsa-miR-223-3p
hsa-miR-551a
hsa-miR-877-3p
hsa-let-7c-5p
hsa-miR-223-5p
hsa-miR-345-5p
hsa-miR-1299
hsa-miR-1304-3p
hsa-miR-193a-5p
hsa-miR-582-3p
hsa-miR-6747-3p
hsa-miR-335-5p
hsa-miR-3613-3p
hsa-miR-206
hsa-miR-215-5p
hsa-let-7b-5p
hsa-miR-542-3p
hsa-miR-145-3p
hsa-miR-6805-5p
hsa-miR-4685-3p
hsa-miR-98-5p
hsa-miR-4646-3p
hsa-miR-204-5p
hsa-miR-1908-5p
hsa-miR-2115-3p
hsa-miR-1255b-5p
hsa-miR-3605-3p
hsa-miR-106b-5p
diff_expr
Count
downregulated
21
not regulated
345
upregulated
38
downregulated
not regulated
upregulated
-log10(PValue)
Log2FC

C)

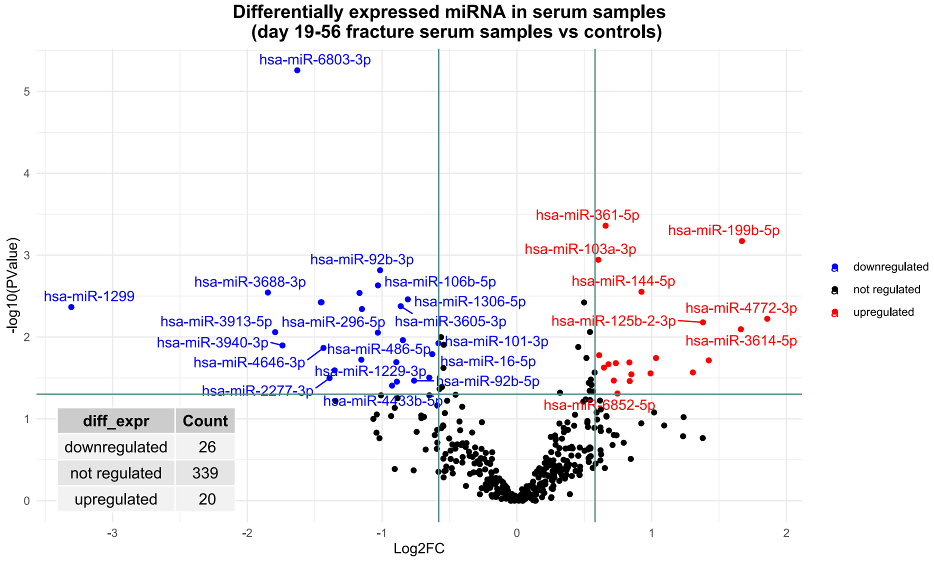

Differentially expressed miRNA in serum samples
(day 19-56 fracture serum samples vs controls)
hsa-miR-6803-3p
hsa-miR-361-5p
hsa-miR-199b-5p
hsa-miR-103a-3p
hsa-miR-92b-3p
hsa-miR-3688-3p
hsa-miR-106b-5p
hsa-miR-144-5p
hsa-miR-1299
hsa-miR-1306-5p
hsa-miR-4772-3p
hsa-miR-3913-5p
hsa-miR-296-5p
hsa-miR-3605-3p
hsa-miR-125b-2-3p
hsa-miR-3940-3p
hsa-miR-101-3p
hsa-miR-3614-5p
hsa-miR-486-5p
hsa-miR-4646-3p
hsa-miR-16-5p
hsa-miR-1229-3p
hsa-miR-92b-5p
hsa-miR-2277-3p
hsa-miR-4433b-5p
hsa-miR-6852-5p
diff_expr
Count
downregulated
26
not regulated
339
upregulated
20
downregulated
not regulated
upregulated
-log10(PValue)
Log2FC
